# A Rare Presentation of Ranula with Supraglottic and Paraglottic Extension Managed via a Hybrid Minimally Invasive Approach

**DOI:** 10.3390/diagnostics16213690

**Published:** 2026-11-09

**Authors:** Jeonghyun Oh

**Affiliations:** Department of Otorhinolaryngology-Head and Neck Surgery, Chosun University Hospital, Gwangju 61453, Republic of Korea; jayoh@chosun.ac.kr

**Keywords:** ranula, paraglottic space, sublingual gland excision, ethanol ablation

## Abstract

A ranula is a mucus extravasation pseudocyst typically arising from the sublingual gland, usually confined to the oral floor or submandibular space. Extension into the supraglottic and paraglottic spaces by traversing or defect-bypassing the mylohyoid muscle is an exceedingly rare phenomenon that poses a significant risk of critical airway compromise. We report an exceptional case of a right-sided ranula presenting with progressive dyspnea due to extensive posterior involvement reaching the vallecula and paraglottic region. Definitive diagnosis of the plunging ranula was established through a comprehensive framework combining clinical presentation, multimodal imaging (CT, MRI, and ultrasonography), histopathological identification of a non-epithelialized lesion with mucinous extravasation, and biochemical fluid analysis via fine-needle aspiration (FNA) confirming markedly elevated levels of salivary amylase and total protein. To mitigate surgical morbidity and avoid extensive transcervical dissection, a hybrid, minimally invasive therapeutic strategy was employed, combining intraoral sublingual gland excision with postoperative percutaneous ethanol ablation for the deep paraglottic residual pseudocystic lesion. Complete resolution of the lesion was achieved without nerve injury, recurrence, or airway complications during follow-up. This case demonstrates that a sequential combination of transoral excision and ethanol ablation is a safe, effective, and organ-preserving alternative to aggressive open neck surgery for deep-extending plunging ranulas.

**Figure 1:**
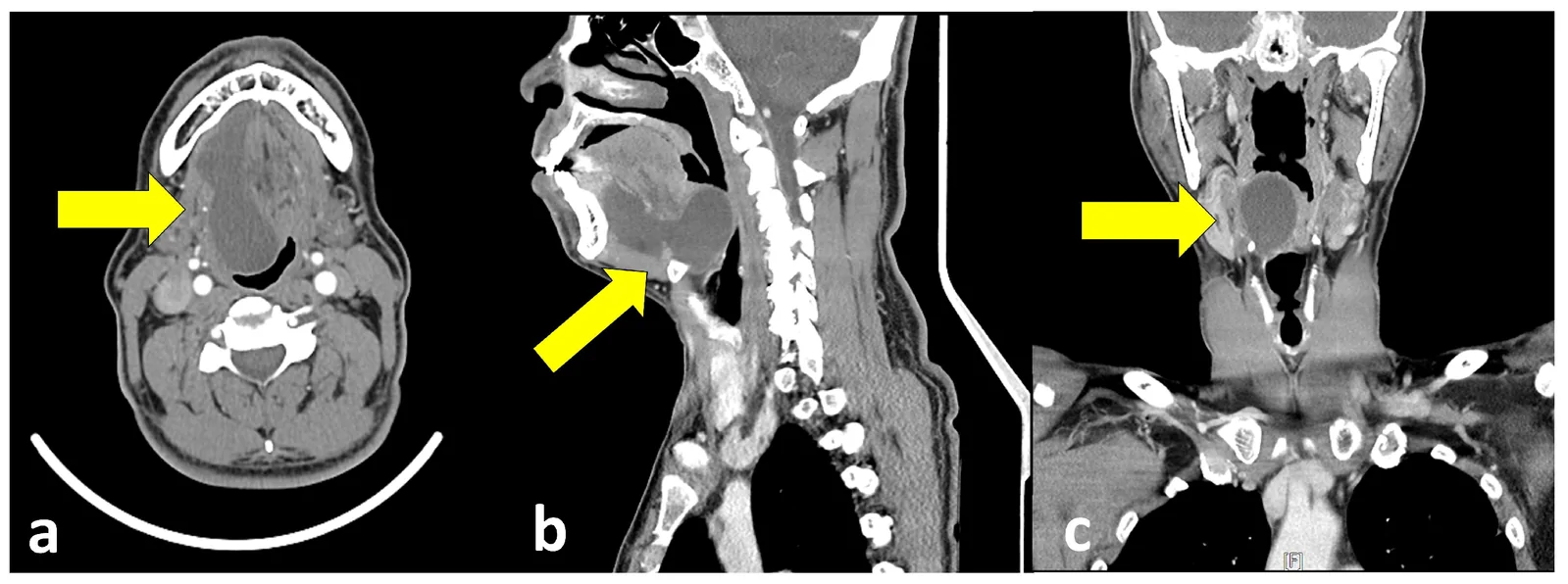
Preoperative neck contrast-enhanced computed tomography (CT) demonstrating a hypodense tubular pseudocystic lesion (indicated by yellow arrows) extending from the right floor of the mouth posteriorly into the tongue base and paraglottic space: (**a**) axial view; (**b**) sagittal view; (**c**) coronal view. Ranulas are mucus extravasation pseudocysts originating predominantly from tears in the sublingual gland ductal system [1]. Although typically confined to the oral floor or submandibular space, deep posterior extension into the paraglottic space presents characteristic imaging features that require distinct radiological differentiation [2]. Both computed tomography (CT) and magnetic resonance imaging (MRI) are essential for delineating the precise anatomical boundaries of these deep plunging lesions extending toward the upper airway [3]. Extensive multi-center clinical reviews confirm that managing complex plunging ranulas often necessitates tailored, multimodal therapeutic strategies beyond simple marsupialization, which carries a high risk of recurrence [4]. A 58-year-old male, with a surgical history of vallecular cyst excision at an outside hospital in 2019, presented with progressive dyspnea and a persistent foreign body sensation in the throat in November 2023. Aside from the prior laryngeal surgery, the patient had no systemic comorbidities (such as hypertension, diabetes mellitus, or cardiovascular disease) and was taking no regular medications, including antiplatelet or anticoagulant agents. To evaluate these symptoms, he underwent an esophagogastroduodenoscopy at the same outside facility, which revealed an oropharyngeal mass. Consequently, he was referred to the Department of Otolaryngology at our hospital under the suspicion of recurrence at the prior surgical site. Flexible laryngoscopy revealed a large, well-demarcated mass involving the tongue base. Cervical CT demonstrated a hypodense tubular lesion extending from the right floor of the mouth to the tongue base, representing a classical presentation of a plunging ranula (Figure 1a–c) [5]. In December 2023, the patient underwent transoral excision of the ranula along with the right sublingual gland under general anesthesia, and histopathological analysis confirmed a mucus extravasation pseudocyst, demonstrating extravasated mucinous pools surrounded by fibrovascular granulation tissue without an epithelial lining. Intraoral sublingual gland resection remains the primary standard treatment aimed at minimizing recurrence rates [6]. During outpatient follow-up from 2023 to 2025, although the tongue base component did not cause recurrent airway obstruction, the residual sublingual lesion exhibited a fluctuating course with intermittent enlargement. Despite conservative interventions, including suture techniques and local steroid injections, the lesion progressively enlarged. A follow-up neck CT in October 2025 confirmed a persistent, localized pseudocystic lesion in the right sublingual space. Given the patient’s strong reluctance to undergo repeat open neck surgery—which inherently carries risks of extensive scarring and potential injury to the marginal mandibular, lingual, and hypoglossal nerves—percutaneous ethanol ablation was selected as an effective, low-morbidity alternative [7].

**Figure 2:**
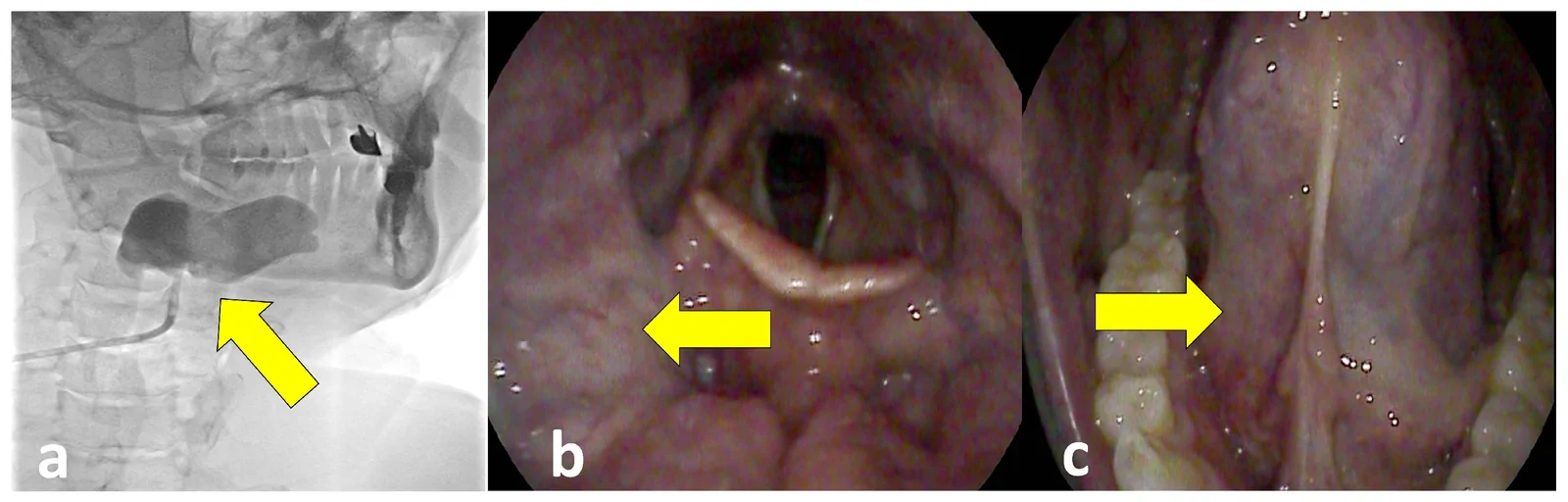
Percutaneous ethanol ablation procedure and subsequent serial follow-up findings: (**a**) Fluoroscopic image obtained during the procedure showing catheter placement and contrast opacification within the submandibular pseudocystic lesion (yellow arrow) prior to absolute ethanol ablation; (**b**) flexible laryngoscopic view at 2 months post-ablation demonstrating marked regression of the airway-compromising mass around the tongue base (yellow arrow); (**c**) flexible laryngoscopic view at 5 months post-ablation showing complete resolution of the fluctuating, large mass in the floor of the mouth (yellow arrow). Under strict real-time ultrasound guidance (USG-guided puncture) combined with fluoroscopy, the right submandibular cavity was targeted using an 8.5 Fr pigtail catheter. Approximately 20 mL of viscous mucoid fluid was aspirated, followed by precise intralesional ablation using 20 mL of absolute ethanol. Ethanol ablation acts through immediate cellular dehydration and severe protein denaturation of the pseudocyst wall, which subsequently promotes secondary fibrotic obliteration. Crucially, ethanol ablation must be conducted with extreme caution and real-time USG monitoring to prevent ethanol extravasation into surrounding neck soft tissues, which can lead to severe complications such as tissue necrosis, nerve injury, or fistula formation into the larynx, hypopharynx, or skin [8]. Immediate post-procedural fluoroscopy verified complete collapse of the lesion, and the catheter was safely removed without acute complications (Figure 2a). Alternative sclerosing agents such as OK-432 have also been utilized for ranula management, supporting the broader utility of minimally invasive sclerotherapy in recurrent cases [9]. At the 5-month follow-up, serial laryngoscopic evaluations showed progressive and sustained size reduction in both the tongue base mass and the sublingual component (Figure 2b,c). The patient remains asymptomatic without evidence of disease recurrence. This case demonstrates that when primary surgical intervention achieves partial airway decompression, subsequent ethanol ablation serves as a safe primary or secondary organ-preserving strategy [10]. A stepwise, multimodal management approach successfully treats deep-extending plunging ranulas while preventing surgical morbidity and neurovascular complications.

## Data Availability

The data presented in this study are available on request from the corresponding author.
